# Supplementary material for: Distinct insulin granule subpopulations implicated in the secretory pathology of diabetes types 1 and 2
Source: eLife. 2020 Nov 9;9:e62506. doi: 10.7554/eLife.62506 (PMC7738183; doi:10.7554/eLife.62506)
Supplement: Figure 2—source data 3. [file elife-62506-fig2-data3.docx]

**Figure 2 – Source Data 3**: Significance values of release durations comparing different cell treatments from Figure 2C.

| **Stimulation** | **Condition** | **Release Duration** | **Number of Cells** | **P-Value** |
| --- | --- | --- | --- | --- |
| 25 mM KCl | untreated | 1.3 ± 0.3 | 5 | 0.6125 |
|  | FFA | 1.4 ± 0.3 | 5 |  |
|  | untreated | 1.3 ± 0.3 | 5 | 0.0121 |
|  | cytokine | 0.78 ± 0.2 | 5 |  |
| 90 mM KCl | untreated | 0.9 ± 0.2 | 5 | 0.0042 |
|  | FFA | 1.4 ± 0.2 | 5 |  |
|  | untreated | 0.9 ± 0.2 | 5 | 0.452 |
|  | cytokine | 0.8 ± 0.2 | 5 |  |

P-values are from simple unpaired Student’s t-tests. They were not corrected for multiple comparisons.
