# Supplementary material for: Distinct insulin granule subpopulations implicated in the secretory pathology of diabetes types 1 and 2
Source: eLife. 2020 Nov 9;9:e62506. doi: 10.7554/eLife.62506 (PMC7738183; doi:10.7554/eLife.62506)
Supplement: Figure 4—source data 2. — Input for Figure 4F and G. [file elife-62506-fig4-data2.docx]

**Figure 4 – Source Data 2**: Table of lipid species, fold change, and significance of the change. Input for Figure 4F and G.

| **Lipid Species** | **ID-Syt9/ID-Syt7 Log2(Fold Change)** | **ID-Syt9/ID-Syt7 -Log2(p-value)** |
| --- | --- | --- |
| DG(16:0/16:0) | 0.11 | 0.47 |
| DG(16:0/16:1) | 0.36 | 0.27 |
| DG(16:0/18:1) | 0.43 | 0.35 |
| DG(16:1/18:1) | 0.31 | 0.23 |
| DG(17:1/16:0) | 0.37 | 0.27 |
| DG(17:1/18:0) | 0.11 | 0.07 |
| DG(17:1/18:1) | 0.56 | 0.44 |
| DG(18:0/16:0) | 0.12 | 1.08 |
| DG(18:0/18:0) | 0.08 | 0.76 |
| DG(18:0/18:1) | 0.32 | 0.29 |
| DG(18:0/20:0) | 0.27 | 1.45 |
| DG(18:0/20:1) | 0.60 | 0.42 |
| DG(18:0/20:3) | 0.54 | 0.54 |
| DG(18:1/18:1) | 0.51 | 0.41 |
| DG(18:1/18:2) | 0.85 | 0.72 |
| DG(18:1/20:4) | 0.62 | 0.51 |
| DG(20:1/18:1) | 0.29 | 0.21 |
| DG(21:1) | -1.55 | 0.77 |
| DG(36:4) | 0.66 | 0.53 |
| DG(38:4) | 0.53 | 0.58 |
| DG(38:6) | 0.61 | 0.49 |
| DG(38:6p) | 0.74 | 0.98 |
| DG(40:5) | 0.57 | 0.55 |
| DG(40:6) | 0.61 | 0.55 |
| DG(40:7) | 0.68 | 0.55 |
| DG(42:5) | 0.01 | 0.01 |
| DG(42:6) | 3.21 | 2.95 |
| DG(44:6) | 0.05 | 0.16 |
| DG(44:7) | 0.00 | 0.00 |
| DG(46:6) | -0.05 | 0.12 |
| DG(46:7) | -0.04 | 0.08 |
| LPC(16:0) | 1.38 | 2.00 |
| LPC(18:0) | 0.93 | 1.22 |
| LPC(18:1) | 0.23 | 0.77 |
| LPC(18:1) | 1.18 | 1.78 |
| LPC(18:2) | 0.15 | 0.42 |
| LPC(18:2) | 0.07 | 0.15 |
| LPC(18:3) | -0.21 | 0.53 |
| LPC(18:3) | -0.11 | 0.19 |
| MG(34:4) | 0.57 | 0.43 |
| PC(15:0/16:0) | 0.03 | 0.11 |
| PC(16:0/16:0) | 0.04 | 0.16 |
| PC(16:0/16:1) | 0.02 | 0.07 |
| PC(16:0/18:1) | 0.02 | 0.05 |
| PC(16:0/20:4) | -0.17 | 0.34 |
| PC(16:1/18:1) | -0.01 | 0.02 |
| PC(18:1/18:1) | 0.00 | 0.01 |
| PC(19:1/16:0) | -0.02 | 0.04 |
| PC(29:0) | -0.10 | 0.19 |
| PC(30:0) | 0.04 | 0.13 |
| PC(30:1) | 0.13 | 0.15 |
| PC(30:1) | 0.11 | 0.37 |
| PC(31:1) | 0.06 | 0.17 |
| PC(32:1e) | 0.15 | 0.59 |
| PC(32:2) | -0.42 | 0.76 |
| PC(32:2) | -0.04 | 0.11 |
| PC(33:1) | 0.00 | 0.01 |
| PC(33:2) | -0.12 | 0.28 |
| PC(34:0) | 0.08 | 0.30 |
| PC(34:1e) | 0.02 | 0.06 |
| PC(34:2e) | 0.14 | 0.42 |
| PC(34:3) | 0.24 | 1.38 |
| PC(34:4) | -0.25 | 0.64 |
| PC(35:2) | -0.01 | 0.02 |
| PC(36:1) | -0.01 | 0.01 |
| PC(36:3) | -0.12 | 0.22 |
| PC(36:4) | 0.08 | 0.19 |
| PC(36:4) | -0.09 | 0.16 |
| PC(36:5) | -0.58 | 1.13 |
| PC(36:5) | -0.04 | 0.08 |
| PC(36:5) | -0.04 | 0.09 |
| PC(36:5) | 0.01 | 0.02 |
| PC(37:2) | -0.01 | 0.01 |
| PC(38:1) | -0.04 | 0.10 |
| PC(38:2) | -0.03 | 0.05 |
| PC(38:3) | -0.24 | 0.43 |
| PC(38:4) | -1.59 | 1.42 |
| PC(38:4) | 0.00 | 0.00 |
| PC(38:4) | -0.15 | 0.27 |
| PC(38:5) | -0.15 | 0.24 |
| PC(38:5) | -0.09 | 0.20 |
| PC(38:5) | 0.02 | 0.05 |
| PC(38:6) | -0.14 | 0.25 |
| PC(38:6) | -0.27 | 0.44 |
| PC(38:6) | -0.11 | 0.18 |
| PC(38:7) | 0.87 | 0.55 |
| PC(38:7) | -0.19 | 0.29 |
| PC(40:1) | -0.21 | 0.37 |
| PC(40:2) | -0.03 | 0.04 |
| PC(40:4) | -0.25 | 0.32 |
| PC(40:5) | -0.17 | 0.23 |
| PC(40:6) | -0.23 | 0.31 |
| PC(40:6) | 0.21 | 0.38 |
| PC(40:6) | -0.21 | 0.33 |
| PC(40:7) | -0.13 | 0.21 |
| PC(42:5) | -0.10 | 0.12 |
| PC(42:6) | -0.14 | 0.19 |
| PE(19:1) | -0.04 | 0.18 |
| PE(19:1) | -0.11 | 0.20 |
| PE(33:0) | -0.02 | 0.05 |
| PE(35:1) | 0.03 | 0.06 |
| PE(35:3) | -0.14 | 0.30 |
| PE(36:1) | -0.10 | 0.24 |
| PE(37:1) | 0.03 | 0.06 |
| PE(37:2) | 0.01 | 0.02 |
| PE(37:4) | 0.05 | 0.12 |
| PE(38:2) | 0.23 | 0.63 |
| PE(38:4) | 0.16 | 0.35 |
| PE(39:1) | 0.00 | 0.01 |
| PE(39:2) | 0.02 | 0.05 |
| PE(39:3) | -0.37 | 0.57 |
| PE(39:4) | -0.19 | 0.30 |
| PE(39:4) | 0.07 | 0.19 |
| PE(39:5) | 0.01 | 0.02 |
| PE(39:6p) | 0.22 | 0.94 |
| PE(40:5) | -0.07 | 0.11 |
| PE(41:2) | 0.06 | 0.09 |
| PE(41:4) | 0.01 | 0.02 |
| PE(41:5) | 0.02 | 0.05 |
| PE(41:6) | -0.38 | 0.58 |
| PE(41:6p) | 0.73 | 1.36 |
| PE(41:7) | -0.19 | 0.29 |
| PE(42:2p) | 1.44 | 1.45 |
| PE(43:5) | 0.02 | 0.03 |
| PE(43:8) | 0.01 | 0.01 |
| PE(45:10) | 0.08 | 0.84 |
| PE(47:10) | -0.07 | 0.25 |
| PE(47:10) | 0.00 | 0.03 |
| PE(47:11) | -0.28 | 2.05 |
| PE(49:13) | -0.20 | 0.56 |
| PE(51:2) | 0.60 | 0.34 |
| PG(35:3) | 0.50 | 1.15 |
| PG(39:3) | -0.06 | 0.24 |
| PG(41:6) | -0.08 | 0.38 |
| PI(34:1) | -0.01 | 0.06 |
| PI(36:1) | -0.02 | 0.12 |
| PI(36:4) | -0.20 | 0.55 |
| PI(38:4) | -0.18 | 0.51 |
| PS(34:0p) | -0.72 | 1.02 |
| PS(36:1) | 0.58 | 0.56 |
| PS(36:1p) | -0.17 | 0.29 |
| SM(d18:1/18:3) | 0.10 | 0.43 |
| SM(d18:1/24:0) | -0.15 | 0.70 |
| SM(d18:1/24:1) | -0.18 | 0.49 |
| SM(d33:1) | 0.12 | 0.75 |
| SM(d34:0) | 0.09 | 0.59 |
| SM(d34:1) | 0.08 | 0.89 |
| SM(d36:1) | 0.07 | 0.43 |
| SM(d36:1) | -0.36 | 1.04 |
| SM(d42:1) | -2.04 | 5.73 |
| SM(d42:1) | 0.04 | 0.14 |
| TG(16:0/14:1/16:0) | 1.06 | 3.10 |
| TG(16:0/16:1/16:1) | 1.44 | 2.57 |
| TG(16:0/18:2/18:2) | -1.40 | 0.94 |
| TG(18:1/18:2/18:2) | -1.75 | 2.01 |
| TG(18:2/18:2/18:2) | -0.19 | 0.11 |
| TG(36:0p) | 0.31 | 0.26 |
| TG(44:11) | -0.01 | 0.03 |
| TG(46:12) | -0.02 | 0.05 |
| TG(54:7) | -0.32 | 0.25 |
