# Supplementary material for: Distinct insulin granule subpopulations implicated in the secretory pathology of diabetes types 1 and 2
Source: eLife. 2020 Nov 9;9:e62506. doi: 10.7554/eLife.62506 (PMC7738183; doi:10.7554/eLife.62506)
Supplement: Figure 5—source data 1. [file elife-62506-fig5-data1.docx]

**Figure 5 – Source Data 1**: Significance values of protein contents between different immunodepleted granules obtained from Figure 5B and Figure 5 – figure supplement 1.

| Protein | Granule Population | Fration of Control | Number of Experiments | P-Value |
| --- | --- | --- | --- | --- |
| syt-7 | syt7 | 0.93 ± 0.07 | 3 | 0.0001 |
|  | syt9 | 0.06 ± 0.01 | 3 |  |
| Doc2B | syt7 | 0.91 ± 0.01 | 3 | 0.0001 |
|  | syt9 | 0.04 ± 0.03 | 3 |  |
| VAMP7 | syt7 | 0.92 ± 0.09 | 3 | 0.0001 |
|  | syt9 | 0.07 ± 0.02 | 3 |  |
| VGAT | syt7 | 0.95 ± 0.04 | 3 | 0.0002 |
|  | syt9 | 0.09 ± 0.1 | 3 |  |
| VNUT | syt7 | 0.89 ± 0.1 | 3 | 0.0001 |
|  | syt9 | 0.04 ± 0.03 | 3 |  |
| MIF | syt7 | 0.86 ± 0.1 | 3 | 0.0005 |
|  | syt9 | 0.1 ± 0.08 | 3 |  |
| IGF2 | syt7 | 0.85 ± 0.08 | 3 | 0.0005 |
|  | syt9 | 0.1 ± 0.1 | 3 |  |
| IAPP | syt7 | 0.85 ± 0.1 | 3 | 0.0003 |
|  | syt9 | 0.09 ± 0.05 | 3 |  |
| PC2 | syt7 | 0.72 ± 0.2 | 3 | 0.0139 |
|  | syt9 | 0.18 ± 0.1 | 3 |  |
| PC1/3 | syt7 | 0.47 ± 0.1 | 3 | 0.4052 |
|  | syt9 | 0.59 ± 0.2 | 3 |  |
| VAMP2 | syt7 | 0.48 ± 0.04 | 3 | 0.2381 |
|  | syt9 | 0.52 ± 0.03 | 3 |  |
| VAMP3 | syt7 | 0.37 ± 0.08 | 3 | 0.3233 |
|  | syt9 | 0.51 ± 0.2 | 3 |  |
| VAMP4 | syt7 | 0.63 ± 0.1 | 3 | 0.5734 |
|  | syt9 | 0.58 ± 0.1 | 3 |  |
| VAMP8 | syt7 | 0.55 ± 0.1 | 3 | 0.0334 |
|  | syt9 | 0.29 ± 0.1 | 3 |  |
| VMAT2 | syt7 | 0.57 ± 0.1 | 3 | 0.0734 |
|  | syt9 | 0.4 ± 0.07 | 3 |  |
| GFP | syt7 | 0.4 ± 0.08 | 3 | 0.0364 |
|  | syt9 | 0.59 ± 0.07 | 3 |  |
| NPY | syt7 | 0.03 ± 0.08 | 3 | 0.0001 |
|  | syt9 | 0.86 ± 0.02 | 3 |  |
| VGLUT1 | syt7 | 0.11 ± 0.06 | 3 | 0.0001 |
|  | syt9 | 0.91 ± 0.05 | 3 |  |
| CAPS2 | syt7 | 0.03 ± 0.03 | 3 | 0.0001 |
|  | syt9 | 0.94 ± 0.07 | 3 |  |
| syt9 | syt7 | 0.09 ± 0.1 | 3 | 0.0002 |
|  | syt9 | 0.98 ± 0.05 | 3 |  |
| sphingomyelin | syt7 | 0.93 ± 0.04 | 3 | 0.0018 |
|  | syt9 | 0.06 ± 0.2 | 3 |  |
| cholesterol | syt7 | 0.74 ± 0.08 | 3 | 0.0029 |
|  | syt9 | 0.26 ± 0.1 | 3 |  |

P-values are from simple unpaired Student’s t-tests. They were not corrected for multiple comparisons.
