# Supplementary material for: Distinct insulin granule subpopulations implicated in the secretory pathology of diabetes types 1 and 2
Source: eLife. 2020 Nov 9;9:e62506. doi: 10.7554/eLife.62506 (PMC7738183; doi:10.7554/eLife.62506)
Supplement: Figure 6—source data 1. [file elife-62506-fig6-data1.docx]

**Figure 6 – Source Data 1**: Significance values between different stimulation strengths for secretion experiments from Figure 6A.

| Secreted Product | Condition | Stimulation | Relative Amount | Number of Experiments | P-Value |
| --- | --- | --- | --- | --- | --- |
| C-peptide-GFP | untreated | 25 mM KCl | 0.39 ± 0.07 | 3 | 0.0008 |
|  |  | 90 mM KCl | 1.00 ± 0.09 | 3 |  |
|  | FFA | 25 mM KCl | 0.5 ± 0.1 | 3 | 1 |
|  |  | 90 mM KCl | 0.5 ± 0.1 | 3 |  |
|  | cytokine | 25 mM KCl | 0.10 ± 0.06 | 3 | 0.0044 |
|  |  | 90 mM KCl | 0.8 ± 0.2 | 3 |  |
| ATP | untreated | 25 mM KCl | 1.06 ± 0.08 | 3 | 0.3574 |
|  |  | 90 mM KCl | 1.00 ± 0.06 | 3 |  |
|  | FFA | 25 mM KCl | 1.2 ± 0.2 | 3 | 0.499 |
|  |  | 90 mM KCl | 1.1 ± 0.12 | 3 |  |
|  | cytokine | 25 mM KCl | 0.26 ± 0.08 | 3 | 0.3233 |
|  |  | 90 mM KCl | 0.4 ± 0.2 | 3 |  |
| Glutamate | untreated | 25 mM KCl | 0.08 ± 0.06 | 3 | 0.0001 |
|  |  | 90 mM KCl | 1.00 ± 0.03 | 3 |  |
|  | FFA | 25 mM KCl | 0.07 ± 0.06 | 3 | 0.3227 |
|  |  | 90 mM KCl | 0.13 ± 0.07 | 3 |  |
|  | cytokine | 25 mM KCl | 0.09 ± 0.1 | 3 | 0.001 |
|  |  | 90 mM KCl | 1.2 ± 0.2 | 3 |  |

P-values are from simple unpaired Student’s t-tests. They were not corrected for multiple comparisons.
