# Supplementary material for: Distinct insulin granule subpopulations implicated in the secretory pathology of diabetes types 1 and 2
Source: eLife. 2020 Nov 9;9:e62506. doi: 10.7554/eLife.62506 (PMC7738183; doi:10.7554/eLife.62506)
Supplement: Figure 6—source data 2. [file elife-62506-fig6-data2.docx]

**Figure 6 – Source Data 2**: Significance values between different treatments for secretion experiments from Figure 6A.

| Secreted Product | Stimulation | Condition | Relative Amount | Number of Experiments | P-Value |
| --- | --- | --- | --- | --- | --- |
| C-peptide-GFP | 25 mM KCl | untreated | 0.39 ± 0.07 | 3 | 0.1936 |
|  |  | FFA | 0.5 ± 0.1 | 3 |  |
|  |  | untreated | 0.39 ± 0.07 | 3 | 0.0055 |
|  |  | cytokine | 0.10 ± 0.06 | 3 |  |
|  | 90 mM KCl | untreated | 1.00 ± 0.09 | 3 | 0.003 |
|  |  | FFA | 0.5 ± 0.1 | 3 |  |
|  |  | untreated | 1.00 ± 0.09 | 3 | 0.1894 |
|  |  | cytokine | 0.8 ± 0.2 | 3 |  |
| ATP | 25 mM KCl | untreated | 1.06 ± 0.08 | 3 | 0.3233 |
|  |  | FFA | 1.2 ± 0.2 | 3 |  |
|  |  | untreated | 1.06 ± 0.08 | 3 | 0.0003 |
|  |  | cytokine | 0.26 ± 0.08 | 3 |  |
|  | 90 mM KCl | untreated | 1.00 ± 0.06 | 3 | 0.2663 |
|  |  | FFA | 1.1 ± 0.12 | 3 |  |
|  |  | untreated | 1.00 ± 0.06 | 3 | 0.0076 |
|  |  | cytokine | 0.4 ± 0.2 | 3 |  |
| Glutamate | 25 mM KCl | untreated | 0.08 ± 0.06 | 3 | 0.8482 |
|  |  | FFA | 0.07 ± 0.06 | 3 |  |
|  |  | untreated | 0.08 ± 0.06 | 3 | 0.8891 |
|  |  | cytokine | 0.09 ± 0.1 | 3 |  |
|  | 90 mM KCl | untreated | 1.00 ± 0.03 | 3 | 0.0001 |
|  |  | FFA | 0.13 ± 0.07 | 3 |  |
|  |  | untreated | 1.00 ± 0.03 | 3 | 0.1619 |
|  |  | cytokine | 1.2 ± 0.2 | 3 |  |

P-values are from simple unpaired Student’s t-tests. They were not corrected for multiple comparisons.
